# Analysis: Flawed Datasets of Monkeypox Skin Images

**DOI:** 10.1007/s10916-023-01928-1

**Published:** 2023-03-18

**Authors:** Carlos Vega, Reinhard Schneider, Venkata Satagopam

**Affiliations:** Bioinformatics Core, University of Luxembourg, Luxembourg Centre for Systems Biomedicine, Av. du Swing 6, Belvaux, 4367 Luxembourg

**Keywords:** Machine learning, Monkeypox, Translational medicine

## Abstract

The self-proclaimed first publicly available dataset of Monkeypox skin images consists of medically irrelevant images extracted from Google and photography repositories through a process denominated web-scrapping. Yet, this did not stop other researchers from employing it to build Machine Learning (ML) solutions aimed at computer-aided diagnosis of Monkeypox and other viral infections presenting skin lesions. Neither did it stop the reviewers or editors from publishing these subsequent works in peer-reviewed journals. Several of these works claimed extraordinary performance in the classification of Monkeypox, Chickenpox and Measles, employing ML and the aforementioned dataset. In this work, we analyse the initiator work that has catalysed the development of several ML solutions, and whose popularity is continuing to grow. Further, we provide a rebuttal experiment that showcases the risks of such methodologies, proving that the ML solutions do not necessarily obtain their performance from the features relevant to the diseases at issue.

## Introduction

Everything began with a pre-print and a skin image dataset aimed to build solutions for computer-aided diagnosis of Monkeypox, Measles and similar diseases. The dataset images were extracted from Google and photography repositories employing web-scrapping techniques and lack any medical validation. This would not have been much of a problem should their dataset and pre-print not have been cited, referenced, or reused in other works, especially in peer-reviewed journals.

In particular, the present paper reviews the dataset and solution presented by Ahsan et al. in the following two pre-prints published in arXiv (see below). However, the crucial aspect discussed hereinafter is how medically irrelevant datasets have been employed and referenced in several subsequent publications, including peer-reviewed articles. Tables 1 and 2 provide a summary of such articles and datasets. From now on, we will refer to the following two pre-prints as the **the initiator work** that has catalysed the development of several ML solutions and other datasets.

- Ahsan, M. M., Uddin, M. R., Farjana, M., Sakib, A. N., Momin, K. A., & Luna, S. A. (2022). **Image Data collection and implementation of deep learning-based model in detecting Monkeypox disease using modified VGG16**. arXiv preprint arXiv:2206.01862 [1].
- Ahsan, M. M., Uddin, M. R., & Luna, S. A. (2022). **Monkeypox Image Data collection**. arXiv preprint arXiv:2206.01774 [2].

Sadly, this issue is becoming more common in the scientific literature. These issues result in data cascades producing a technical and scientific debt that becomes harder to correct as time passes [3]. We believe that the problem addressed in this paper could have been easily avoided by conducting internal review prior to the pre-print publication. Further, the entire quality control chain of the scientific literature seems to have failed. The issue reaching peer-reviewed journals could have been prevented by conducting thorough reviews on the works during the submission process. We believe that the responsibility does not necessarily lay on the authors of the original dataset entirely, who upon contact clarified their role as graduate students. Instead, their managers in line should have controlled and reviewed the work to prevent the dataset from spreading. All in all, the quality of scientific literature is everyone’s responsibility.

**Table 1:** Examples of peer-reviewed and pre-print articles referencing the original work, sorted by publication date

**Title**	**Journal**	**Peer-reviewed**	**Date**
Can Artificial Intelligence Detect Monkeypox from Digital Skin Images? [25]	BiorXiv	No	Aug. 2022
Current and Perspective Sensing Methods for Monkeypox Virus [26]	MDPI Bioengineering	Yes	Oct. 2022
Human Monkeypox Classification from Skin Lesion Images with Deep Pre-trained Network using Mobile Application [27]	Springer Journal of Medical Systems	Yes	Oct. 2022
Monkeypox Virus Detection Using Pre-trained Deep Learning-based Approaches [28]	Springer Journal of Medical Systems	Yes	Oct. 2022
Artificial intelligence (AI) in Monkeypox infection prevention [29]	Journal of Biomolecular Structure and Dynamics	Yes	Oct. 2022
Meta-Heuristic Optimization of LSTM-Based Deep Network for Boosting the Prediction of Monkeypox Cases [30]	MDPI Mathematics	Yes	Oct. 2022
Convolutional Neural Network for Monkeypox Detection [31]	Springer Lecture Notes in Networks and Systems	Yes	Nov. 2022
Classification of Human Monkeypox Disease Using Deep Learning Models and Attention Mechanisms [32]	arXiv	No	Nov. 2022

**Table 2:** Summary of datasets

**Title**	**Journal**	**Peer-reviewed**	**Date**
Monkeypox Image Data collection [2]	ArXiv	No	Jun. 2022
Monkeypox Skin Lesion Detection Using Deep Learning Models: A Preliminary Feasibility Study [33]	ArXiv	No	Jul. 2022
A Web-scrapped Skin Image Database of Monkeypox, Chickenpox, Smallpox, Cowpox, and Measles [34]	BiorXiv	No	Aug. 2022

### Context and recent precedents

During the COVID-19 pandemic, several researchers rushed to develop solutions for the diagnosis of COVID-19 by employing X-Ray images. The solutions and datasets developed during the initial stages of the pandemic broke all the rules and guidelines regarding ethical data science and proper scientific methodology [4–6]. Months later, several works were published criticising and highlighting the pitfalls and mistakes committed during the pandemic. Roberts et al. provided a systematic review for 62 studies employing Machine Learning models for the diagnosis or prognosis of COVID-19 from chest radiographs and chest computed tomography images. The authors concluded that none of the models were adequate for potential clinical use due to methodological flaws and/or underlying biases [4]. Similarly, Garcia et al. conducted a systematic review of the datasets employed in works designed for computer-aided-diagnosis and stratification of COVID-19 based on chest radiographs. They found just 9 datasets out of more than a hundred meeting the criteria for proper assessment of risk of bias and concluded that the most popular datasets used in over two hundred peer-reviewed articles did not include these 9 datasets [7].

All these issues are not new, but events such as pandemics draw interest from the scientific community, producing an explosion of manuscripts and works on the topic, amplifying the issues in turn. In the case of COVID-19, during the first seven months of 2020, 30,000 coronavirus-related papers were published, from which over 1,000 included the terms “machine learning”, “artificial intelligence”, “deep learning”, or “neural network” in the title or abstract [8]. Regarding Monkeypox literature, more than 1,400 articles have been published since the beginning of 2022 [9].

#### Quote 1: From the original article by Ahsan et al. arXiv:2206.01862

“Our motivation in establishing the Monkeypox data set is inspired by Dr Joseph Cohen, who generated the dataset during the onset of COVID-19 by gathering the dataset from numerous sources, including websites and papers.”

The image repository discussed in this paper (see Quote 1) was created based on a similar repository created by Dr Joseph Cohen in 2020 [11]. Cohen’s repository was built to gather images of X-Ray scans of COVID-19 patients. The authors of the Monkeypox dataset [12] were inspired by this work to create a similar repository for Monkeypox skin images. However, the authors do not mention the number of papers and literature criticising the approach of Dr Cohen and the solutions developed with it. The concerns included poor labelling, incoherence, bias, data acquisition variety, patient consent, etc. Nevertheless, after the wave of critics, Dr Cohen included the following note in the repository: “please do not claim diagnostic performance of a model without a clinical study! This is not a Kaggle competition dataset”. Additionally, Dr Cohen has dedicated several efforts to study the issues and avoiding the problems reported in the literature, including the organisation of seminars, talks and designing a protocol for his repository, approved by University of Montreal’s Ethics Committee [13].

**Fig. 1:**
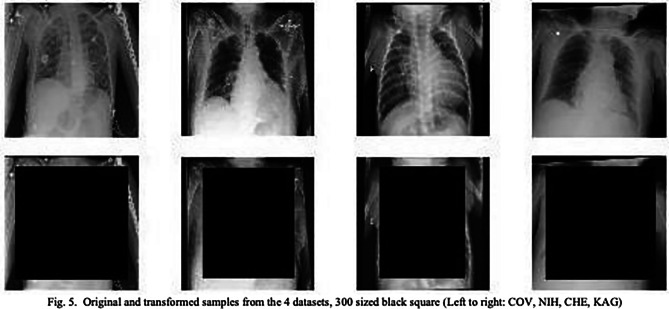
Blinding the dataset from the supposed relevant features. Image extracted from the work of Maguolo et al. [10]. Our rebuttal experiment follows a similar approach as shown in Fig. 2

**Fig. 2:**
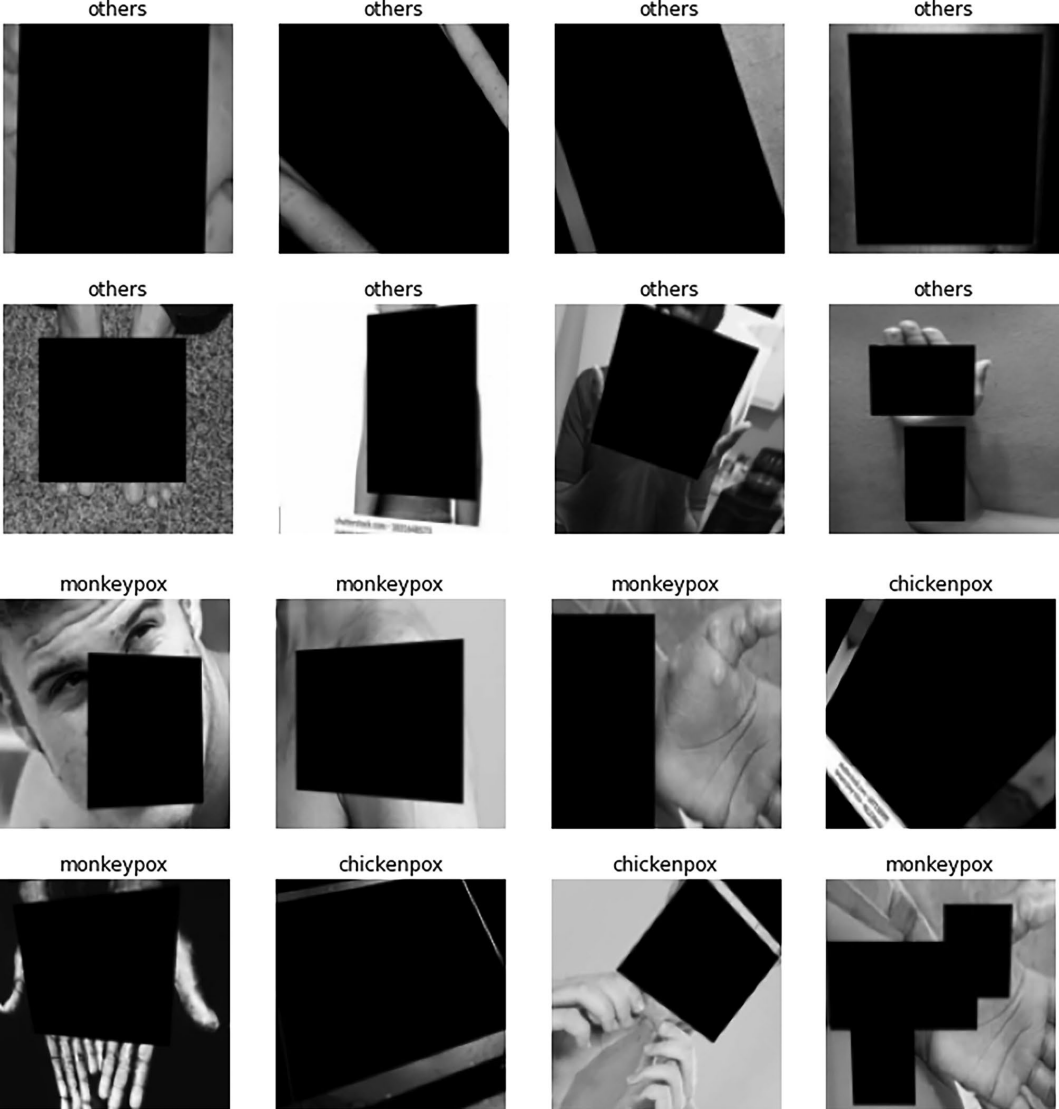
Batch of *blinded* images. Each image includes their respective label

Soon after the release of Dr. Cohen repository, several works found that such repository suffered from several issues that led to biased Machine Learning solutions [10, 14]. And it is precisely the work of Maguolo et al. which inspired the rebuttal experiment of the present paper. Figure 1 presents the approach employed to showcase the weaknesses of several Machine Learning solutions developed with chest radiographs of COVID-19 patients. Our proposed rebuttal experiment follows a similar approach as depicted in Fig. 2.

### Structure of the paper

In the remaining of this paper we present an analysis (see “Analysis of the initiator dataset and ML solution” section) highlighting the risks and malpractices derived from the papers at issue. “Methods of the rebuttal experiment” section provides a rebuttal experiment to exemplify the issues of the dataset and ML solution. “Impact and similar works” section describes the impact of this initial dataset and pre-print, which has become popular in several pre-print works and peer-reviewed publications. Finally, “Discussion” section briefly discusses different sets of good practices to avoid the issues identified in this work.

## Analysis of the initiator dataset and ML solution

**Fig. 3:**
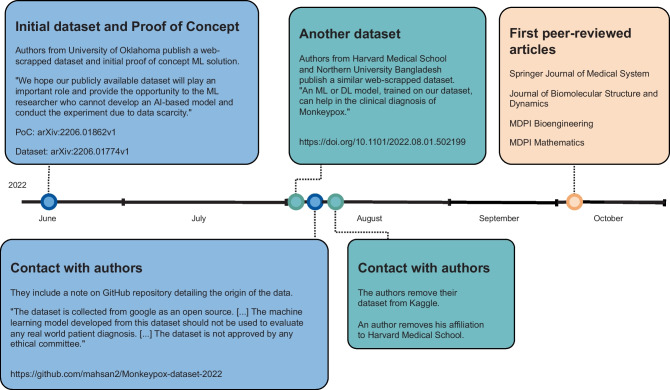
Overview of the publication timeline of the original paper and dataset and subsequent works

In the following section we analyse the data acquisition details and composition of the initiator work. Namely, the initial dataset together with the results of the Machine Learning solution developed by Ahsan et al. [1, 2]. Figure 3 summarises the time evolution of the initial work and subsequent works.

### Description and work methodology

The pre-print paper published in arXiv claims to identify Monkeypox patients with an accuracy of 97%. The motivation of the paper is clear: to diagnose Monkeypox employing images of the skin. However, there are several concerns regarding whether the provided dataset and the ML solution help to achieve this goal.

The authors present two “studies”:

- Study one: Aims to classify between Monkeypox and Chickenpox.
- Study two: Aims to classify between Monkeypox and others.

The authors argue:

#### Quote 2: From the original article by Ahsan et al. arXiv:2206.01862

“Healthcare professionals can easily adapt our model as it is cost and time effective and does not require extensive PCR or microscopy testing. As an effect, our proposed model provides an opportunity to test in real-time screening of the patients with Monkeypox symptoms.”

We believe the authors did not assess whether the model was actually modelling the diseases and not just distinguishing the classes based on any other surrogate features present in the images. As explained before, there were similar concerns with several COVID-19 solutions published during the pandemic.

The present analysis hypothesises that the trained classifier is not learning to identify the diseases but rather, to separate the two sets of images. Therefore, it is not learning the phenomena or any feature related to the diseases. To test this hypothesis, the dataset was “blinded” by adding rectangles over the regions of interest in the images (see Fig. 2), which are presumed to be the blisters, pustules and rash areas. Again, this approach is similar to the one employed by Maguolo et al. to assess the performance of COVID-19 solutions [10] (see Fig. 1).

At first glance, the initiator work presents a number of methodological issues that we summarise below.

- The authors did not share the employed code. However, they shared enough details to replicate their results.
- The authors provided a poorly collected dataset (web-scrapped from Google) with samples from Monkeypox, Chickenpox, Measles and Normal.
- The images provided have not been reviewed or curated by medical experts.
- The authors state to include images from infected patients, but no information is provided regarding participant’s consent or ethical review board.
- Some of the images included in the dataset are licensed.
- Class imbalance and data augmentation are not properly handled.
- The work lacks external validation or stratified performance assessment.

The rest of the analysis delves into each of these issues in detail.

#### Data acquisition process

The authors did not utilise any medical dataset, arguing that no public dataset was available yet. Rather, they collected samples from Google, purposely selecting images with commercial rights, as described in the first figure of the original paper. In consequence, the dataset contains licensed images from Getty Images, Shutterstock, Dreamstime and other stock image repositories. A couple of examples can be inspected in [15, 16]. The authors collected 43 images labelled as Monkeypox, 47 for Chickenpox, 17 for Measles, and 54 images labelled as Normal. These images lack any coherence or protocol regarding the framed view, modality or body parts photographed.

The data acquisition procedure of this dataset should be enough reason to avoid employing the dataset in any relevant work aimed at computer-aided diagnosis. Still, the authors state:

##### Quote 3: From the original article by Ahsan et al. arXiv:2206.01862

“We hope our publicly available dataset will play an important role and provide the opportunity to the ML researcher who cannot develop an AI-based model and conduct the experiment due to data scarcity.”

The article claims that the predictions of the model were cross-checked by doctors (see Quote 4). However, the authors did not describe the process or how the images were assessed. Therefore, it is not possible to confirm or replicate the verification process. This is an important part of the paper that is completely missed. However, since this work is a pre-print, we could expect future versions to include further details. Nevertheless, the work was employed *as is* by other subsequent research works, including peer-reviewed works.

##### Quote 4: From the original article by Ahsan et al. arXiv:2206.01862

“Our data collection procedure and model performance are analyzed by expert doctors who ensure our model’s satisfactory performance.”

Moreover, **the authors state that they present a “dataset containing Monkeypox infected patients”**. This raises several questions. First, it is not clear whether the provided images stem from real patients. If so, did such patients consent to provide the images to the University of Oklahoma for this purpose? And finally, were the diagnosis actually corroborated through any other medical test? In these regards, the manuscript does not mention the participation of ethical boards from the University of Oklahoma or other affiliated institutions during the development of their research.

#### Licensing of google search and stock image repositories

The paper mentions that the images were collected using google’s “free to use even for commercial purpose” option. The dataset contains licensed images from several repositories such as Getty Images, Shutterstock and Dreamstime (among others). Moreover, the first figure of the article shows that the option “Commercial & other licenses” was selected in Google Search to collect the images. The dataset does not include meta-information indicating the origin of the images. Two example of these images can be accessed in the following references [17, 18] that can be found in the Github repository of the dataset [15, 16], respectively.

Below we provide the meaning of “Commercial or other licenses” from Google and the licensing statements from some image repositories.

##### Quote 5: Google, [19]

“Commercial or other licenses: These images have non-Creative Commons licenses and can be from either sites available at no charge or commercial sites that require payment.”

##### Quote 6: Dreamstime, [20]

“Conditional upon your compliance with this Agreement, Dreamstime grants you a limited license to download Watermarked Media solely for evaluating/comping whether you wish to purchase a license to the Non-Watermarked Media according to the Standard Terms and Conditions applicable to your use. You may not use a Watermarked Media in any final materials distributed within your company or any materials distributed outside your company or to the public or in any online or other electronic distribution system.”

##### Quote 7: Getty Images, [21]

“Getty Images: Using images for free. The images on Getty Images are intended for use in commercial and editorial projects. This means you need to buy a license to use the image in most projects, including personal use. You can use an image without paying for a license with our Embed feature, which lets you use over 70 million photos on any non-commercial website or blog (if you’re using it to sell a product, raise money or promote or endorse something, Embed isn’t for you). Just do a search, then go to Filters to turn on the Embeddable images filter on the search results page.”

Of course, regardless of the licensing issues from watermarked media, **images from stock repositories are not a reliable source of medically validated images**. Specially if the solution is designed to help diagnosing Monkeypox, Measles, and Chickenpox employing such images as representative examples of these diseases. Moreover, this data acquisition process precludes obtaining further information such as a diagnosis corroboration via other tests or demographic information to help assess the sample diversity and solution performance in different subgroups.

#### Data augmentation

The paper does not clarify whether the data augmentation was performed in a way that prevents data leakage of augmented instances into the valid/test set. Often, data augmentation entails a previous step to the training process to increase variability of the dataset, but the augmented images are not meant to be preserved or shared in order to prevent any potential misuse. For instance, data augmentation can lead to data leakage, e.g., if the variations of an image are mixed across test and train sets. The paper does not provide information to tell how data was managed or split, e.g., group-wise split to prevent breaking the independent and identically distributed assumption. This issue is also known as row-wise leakage. This is one of the reasons that motivated us to contact the authors by e-mail requesting the original code. Unfortunately, the authors did not provide the original code. However, they clarified that their work was just part of their training process as graduate students.

#### Class imbalance

The authors acknowledge the class imbalance in their work but do not re-balance the dataset in any way, for instance, augmenting just the imbalanced classes. There is no mention of any other measure to tackle the class imbalance. Also, there is no further information, such as demographic information, that could allow for a stratified evaluation of the ML model performance with respect to different subgroups (e.g. ethnicity, colour skin, age, gender).

#### External validation

The authors do not provide any external validation. Their validation set consists of a split of images from the same dataset, which has the same generative process as the rest of the dataset. A tool aimed to conduct clinical diagnosis should be thoroughly evaluated against different external datasets to increase the confidence on the model. Otherwise, chances are, that the built classifier just fits the dataset, without actually learning the underlying phenomena intended to model. Therefore, there is no proof of any verification by experts of this initial web-scrapped dataset or proper external validation. In “Discussion” section, we further delve into this issue.

### Methods of the rebuttal experiment

This section describes the methods employed to conduct the rebuttal experiment aimed to test the hypothesis of the present manuscript. Namely, that the models’ performance of the initiator work does not stem from proper modelling of the underlying phenomena but rather shows an overfitting performance illusion of the dataset.

Importantly, this rebuttal experiment purposely avoids fixing the class imbalance issues to resemble the original work. To test the hypothesis of this analysis, i.e., whether the model is learning the features relevant to the disease, the dataset images were *blinded* by adding rectangles over the regions of interest of the images, which are presumed to be the blisters, pustules and rash areas. Examples are shown in Fig. 2.

The experiment from the initiator paper was replicated employing Python and using the libraries Fastai 2.7.8 and Tensorflow 2.5.0 employing GPU NVIDIA Tesla V100-SXM2-16GB from the Iris cluster [22] of the High Performance Computing facilities of the University of Luxembourg. Likewise the deep learning (DL) solution from the original paper, a Visual Geometry Group (VGG) convolutional neural network (CNN) with 16 layers was employed [23], i.e., VGG16_BN [24] from torch vision library. BN stands for batch normalization. A summary of the code can be found in Fig. 6 in Appendix.

### Results

**Fig. 4:**
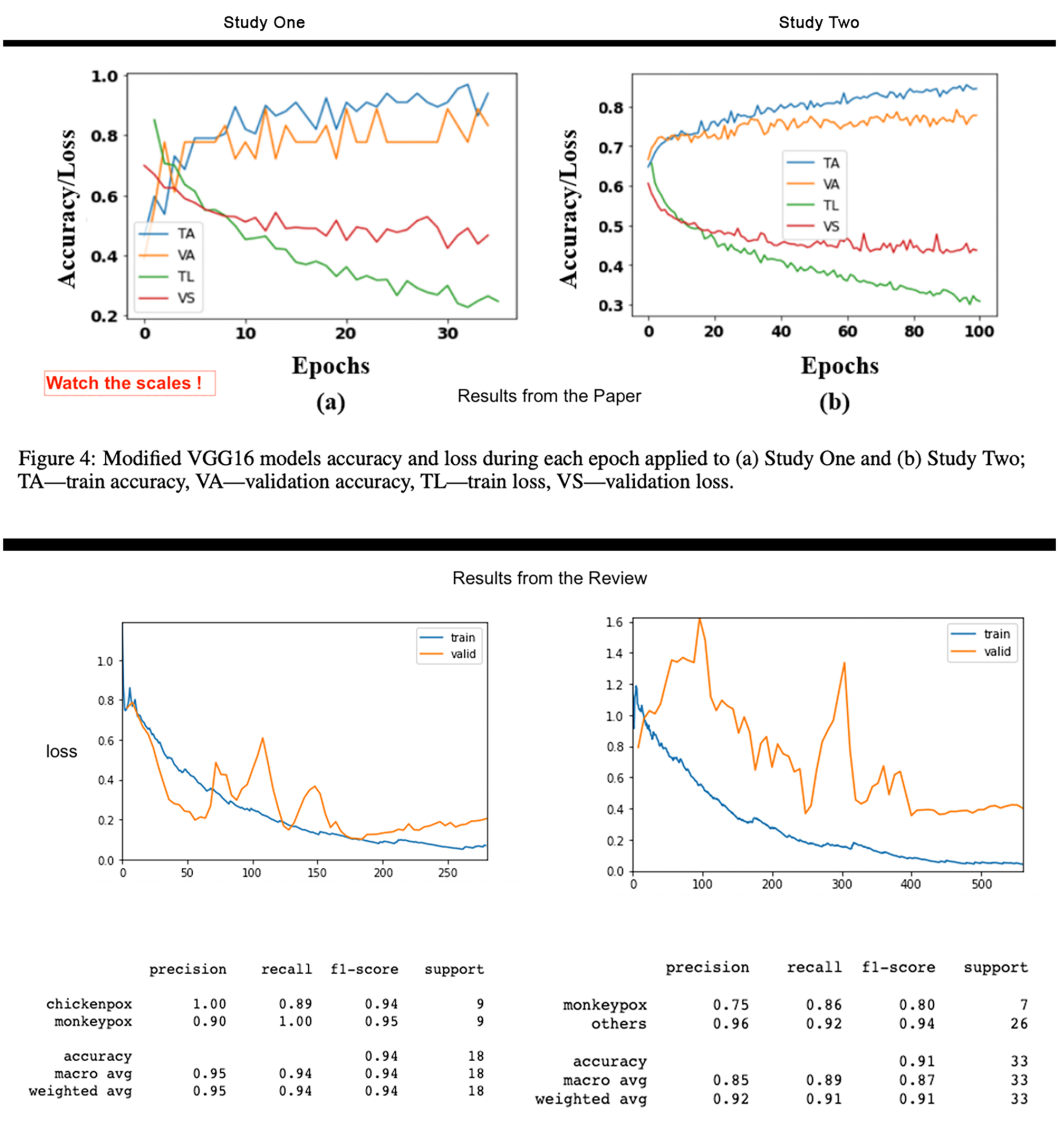
This figure depicts the original results in the top half for both study one and two, taken from the original paper. On the bottom, the results of this analysis indicating a similar performance

The resulting model can accurately classify the given classes in both studies (i.e., Monkeypox vs Chickenpox and Monkeypox vs others) even though the relevant areas of the images have been *blinded*. Therefore, we can conclude that the model built by Ahsan et al. is not properly modelling the phenomena they try to model. In consequence, such solution is not suited for clinical diagnosis, neither the dataset is recommendable for any research on medical Machine Learning. The conclusions of this rebuttal experiment can be extended to other works following a similar approach with similar datasets and methods (see “Impact and similar works” section). Figure 4 compares the results.

The original work claimed to identify Monkeypox patients with an accuracy of 97% and 88% for studies one and two, respectively. Figure 4 shows a similarly good general accuracy of 94% and 91% and precision for Monkeypox of 90% and 75%. More metrics are provided because in highly imbalanced datasets the accuracy alone is not representative of the model’s performance. Again, the dataset neither provides metadata nor demographic information that could allow to conduct a stratified performance evaluation of the models.

## Impact and similar works

At this point, none of this may seem much of a problem. After all, the authors’ paper has not been published in any scientific journal or conference proceedings yet. However, several subsequent works have based their research upon their work and dataset. It is now common to cite and reference papers published in publication repositories, pre-print servers and other non-reviewed publication services. Their dataset remains open and available for the potential misuse of the research community.

**Sadly, some of the works employing this dataset have been published in peer-reviewed journals and conferences**. A summary is provided in Table 1.

### Pre-print articles

In August 2022, the preprint “A Web-scraped Skin Image Database of Monkeypox, Chickenpox, Smallpox, Cowpox, and Measles” was uploaded to BiorXiv publication repository. In this case, one of the authors is affiliated with Boston Children’s Hospital, Harvard Medical School [34]. At first glance, it is easy to wonder whether the diagnosis relevance of the images was validated by medical doctors or not. After further enquiries and contacting the authors, they clarified that the author was personally helping some of the other authors with a graduate project and that they mistakenly included his affiliation on the pre-print manuscript without his consent. The same authors also published a Kaggle dataset, which is referenced in their pre-print and available as part of their supplementary material. This dataset was taken down from Kaggle after contacting the authors with further interest on the validity of their dataset. Such dataset suffered from similar methodological issues. Namely, the images lacked coherence regarding the modality or point of view, i.e., some of them were close up images from the skin, other included hands, mouths, faces etc. The paper claims that “An ML or DL model, trained on our dataset, can help in the clinical diagnosis of Monkeypox”. However, proofs on these regards are not provided.

In November 2022, another group of authors from United International University published a pre-print employing the same kind of datasets and approaches. Similar works are spreading in the form of pre-prints, increasing the citations and popularity of the initial works [32].

### Peer-reviewed articles

In October 2022, Springer Journal of Medical Systems published two peer-reviewed articles referencing the initator work. The first of these articles, entitled “Monkeypox virus detection using pre-trained deep learning-based approaches” [28], employs the aforementioned dataset, stating that:

#### Quote 8: From Sitaula et al. 2022, Journal of Medical Systems

“More recently, Ahsan et al. [1] collected the images of Monkeypox, Chickenpox, Measles and Normal categories using web mining techniques and verified by the experts”.

Again, there is no proof of any verification by experts of this web-scrapped dataset. The authors Sitaula et al. also state that “These encouraging results, which outperform the state-of-the-art methods, suggest that the proposed approach is applicable to health practitioners for mass screening”. However, the authors have not validated their solutions’ performance in real-world settings. According to Google Scholar, by February 2023 this article had 14 citations.

The second article from Springer Journal of Medical Systems entitled “Human Monkeypox Classification from Skin Lesion Images with Deep Pre-trained Network using Mobile Application” [27] references the initator work, but employs a different dataset to develop their Mobile Application. In particular, their dataset was obtained from the work conducted by Ali et al. [33] who published it in Kaggle [35]. However, once again, the acquisition process of this dataset does not present a clear protocol for the validation of the images as stated in Quote 9. Again, this dataset was acquired employing a manual web-scrapping approach.

#### Quote 9: From Ali et al. 2022, arXiv:2207.03342

Our monkeypox skin lesion dataset is primarily compiled from publicly available case reports, news portals, and websites through extensive manual searching. We did not use automatic web-scrappers. [...] All the skin lesion images were verified using Google’s Reverse Image Search and cross-referenced with other sources. Through a 2-stage screening process, the out-of-focus, low-resolution, and low-quality images were discarded, and only the unique images that satisfy the quality requirements were selected.

Another paper entitled “Current and Perspective Sensing Methods for Monkeypox Virus” was published in October 2022 in MDPI Bioengineering [26]. This work indicates that “the images used in the dataset are from previous outbreaks” when referring to the original dataset published by Ahsan et al.

The work “Artificial intelligence (AI) in Monkeypox infection prevention”, published in October 2022 in the Journal of Biomolecular Structure and Dynamics [29], appropriately refers to the dataset as “the first public Monkeypox image dataset by collecting images from a variety of sources (e.g. newspapers, websites)”. However, it does not delve into the medical relevance of the images.

Finally, the article “Meta-Heuristic Optimization of LSTM-Based Deep Network for Boosting the Prediction of Monkeypox Cases”, published in MDPI Mathematics in October 2022, refers to the dataset as: “developed by gathering photos from various open-source and internet resources, providing a safer approach to utilizing and disseminating such data for developing and deploying any ML model. [...] This model’s prediction and feature extraction helped to provide a deeper insight into specific features of the Monkeypox virus”. In this case, the data acquisition workflow was properly described, however, there are no further details regarding what specific insights into the features of Monkeypox they refer to.

## Discussion

The works tackled in this manuscript suffer from issues on several fronts, from data acquisition to external validation, passing through data curation and solution design. However, we should not ignore the fuel that boosts these issues, which is strongly tied to the current scientific publication culture that pressures researchers to publish more and faster [36, 37], leading to a literature full of inaccurate works and disinformation for which biomedical imaging research is no exception [38, 39].

In the remainder of this section, we superficially tackle different good practices for the aforementioned stages since the literature already provides an extensive record of guidelines, checklists and recommendations for different use cases and tasks [40–42].

### Data-centric AI

Nobody questions the huge opportunities that Artificial Intelligence (AI) and ML bring to bioinformatics and computer-aided diagnosis [43], but these opportunities come with challenges [44–47]. The first is data, which needs to adhere to high-quality standards that vary from area to area. Thus, there is no golden rule suiting all use cases, but there are common practices worth noting regarding data collection.

#### Domain knowledge

Producing high-quality data requires incentives and **involvement of domain experts** [3]. For instance, AI for medical imaging in radiology needs radiologists’ feedback and participation in the data annotation process [48]. Similarly, Goyal et al. highlight the importance of communication between AI specialists and dermatologists in the development of solutions for skin cancer diagnosis [49]. AI solutions for dermatological tasks require careful data design and accurate data annotation to prevent performance issues [50]. Liang et al. note that “a systematic assessment of three computer AI models for diagnosing malignant skin lesions demonstrated that the models all performed substantially worse on lesions appearing on dark skin compared with light skin” [51, 52]. These performance disparities did not have a single cause. The authors note that changing the way the models process the dataset images did not significantly improve the performance, but improving the annotations and skin sample diversity produced the desired performance jump. Hutchinson et al. provide a rigorous framework for dataset development [53] highlighting the importance of a non-linear dataset life-cycle.

#### Sample representativeness

Another crucial aspect concerns all the additional information that despite not being used to train the ML models may become indispensable to assessing the performance and generalisation power of the model. ML performance is often reported through metrics such as accuracy, precision or F1 score that summarise the overall performance of the model without giving further details on their generalisation capabilities. As stated by Garcia et al., common ML safe practices like cross-validation or class imbalance control can minimise model issues like over-fitting. However, “their use draws from the premise that data is a solid representation of the modelled phenomena”. Hence, these practices cannot overcome data collection issues [54]. For instance, demographic information such as ethnicity, age, and gender may not be provided to the classifier, but anyway used to ensure that the performance is acceptable across different *stratas* (combinations of age groups, gender, ethnicity, etc.). Thus, such information becomes key to assessing the sample representativeness. Goyal et al. note the performance discrepancies in ML models for diagnosis of skin cancer across datasets with poor skin tone diversity. Moreover, they emphasise the importance of inter-class similarities in skin lesions which may lead to poor classification performance in ML models trained with small and unvaried samples [49], calling for more granular classes in classification problems. All in all, data acquisition and curation of representative data has been identified as the most common way to mitigate model biases [55].

#### The importance of metadata

Similarly, **metadata information** provides information about the acquisition details. DICOM files [56] may include information regarding the modality, position, machine brand, etc. that allow for performance assessment and data exploration with respect to the acquisition tools and protocols employed. Garcia et al. share a classical radiology example that may have counterparts in other domains. Different imaging protocols may be employed depending on the patient’s health status. For example, “if the patient is bed-bound the clinical staff is forced to carry out AP (anterior-posterior) supine imaging” with a portable device, instead of the preferred PA (posterior-anterior) prone protocol. Thus, an ML system could potentially associate features present in PA images with better diagnostic and prognostic outcomes [7].

Thus, metadata can help identify these issues beforehand during the exploratory data analysis and assess the model’s performance across different variables. In the case of image photographs, information regarding the white balance may help normalise or correct the illumination and lighting effects caused by a light source during image acquisition [49]. Moreover, **causal information** can enable meta-comparison of data acquisition pipelines providing better reproducibility and replication of the solution development from the data acquisition to the algorithm training. In their recent paper, Garcia et al. share a series of guidelines for safer data-driven ML solutions through actionable causal information and metadata approaches [54]. Namely, the authors argue that the inclusion of causal information in the data generation process can help prevent confounding effects while metadata information eases dataset auditing and model evaluation.

#### Multi-modal data

Medical diagnosis is inherently multi-modal, requiring multiple lines of evidence and varied tests. This fact calls for multi-modal data in computer-aided diagnosis too, which is especially important given that one disease can have more than one aetiology, and one aetiology can lead to more than one disease [57]. Goyal et al. share similar advice, noting the importance of other sources of evidence like biopsies to confirm the diagnosis, but also point out the risks posed by heterogeneous data sources, which produce noisy data for which machine learning algorithms are particularly sensitive [49].

To sum up, better documentation of the data acquisition process, causal assumptions, data annotation and curation protocols, help to mitigate and identify potential sources of bias and prevent data cascades [3, 58]. All of it, in pursuit of more robust ML models.

### External validation

Assessing the performance of an ML system with respect to a particular dataset does not suffice to assume a similar performance in a different setting or data distribution. The population characteristics may differ from region to region and country to country, including comorbidities, disease incidence and demographics. Thus, a model may require calibration or re-training before being deployed in a new setting. Before employing a prediction model, it is crucial to assess the predictive performance in datasets that were not used to develop the model. A systematic review conducted by Collings et al. on medical prediction models, states that “the majority of published prediction models are opportunistic and are rarely being used or even mentioned in clinical guidelines” [59]. Their results report that out of 78 articles eligible for review, 33 (42%) described an evaluation on a separate dataset, but 12 of them employed data from the same centre at a different time-period for temporal validation.

External validation is often designed on a case-by-case basis, but there are general tips and methods worth mentioning. Cabitza et al. propose a meta-validation approach to assess external validation procedures in medical ML models [60]. The method encompasses two steps and a series of visualization aids to interpret the results. The first step entails calculating an estimate of robustness in terms of performance dependence on the similarity between training and test sets. This step does not require an external dataset. Conversely, the second step does require external datasets to assess the performance in terms of similarity and cardinality with respect to the training set. This method helps to assess the diversity of external datasets with respect to the training set.

In this sense, external validation is a strong motivation to build public resources with high-quality dataset repositories, which again require incentives and funding to promote data curation. After all, finding datasets for external validation is undeniably hard. For instance, the solution from Rizk et al. for Meniscal lesion detection employed the MRNet Dataset from Stanford ML group, a dataset seven times smaller than the main dataset [61, 62]. In another work, Faes et al. employed five open-source datasets to develop five corresponding models of different kind but they could only conduct external validation on one of them, stating that “the external validation of these models was insufficient” [63]. Han et al. note the limitations of the external validation conducted for their proposed neural network algorithm for the diagnosis of skin neoplasms, “the Edinburgh dataset consists primarily of data corresponding to white subjects” [64].

### MLOps

**Fig. 5:**
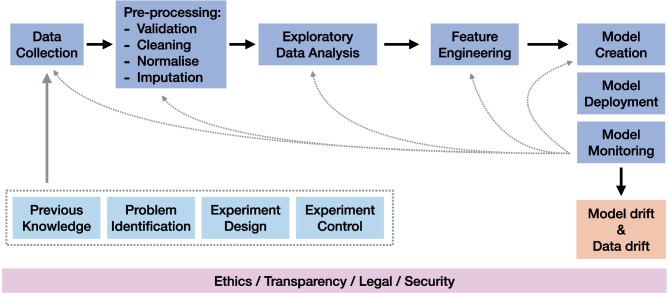
Simplified MLOps workflow. Light blue boxes represent *prior* considerations that condition data collection. Similarly, ethics and regulations (pink) affect the whole pipeline. In practice, each stage is prone to feedback information to the previous stage for new iterations, but for the shake of simplicity the diagram highlights the impact of Model Monitoring in the whole pipeline

**Fig. 6:**
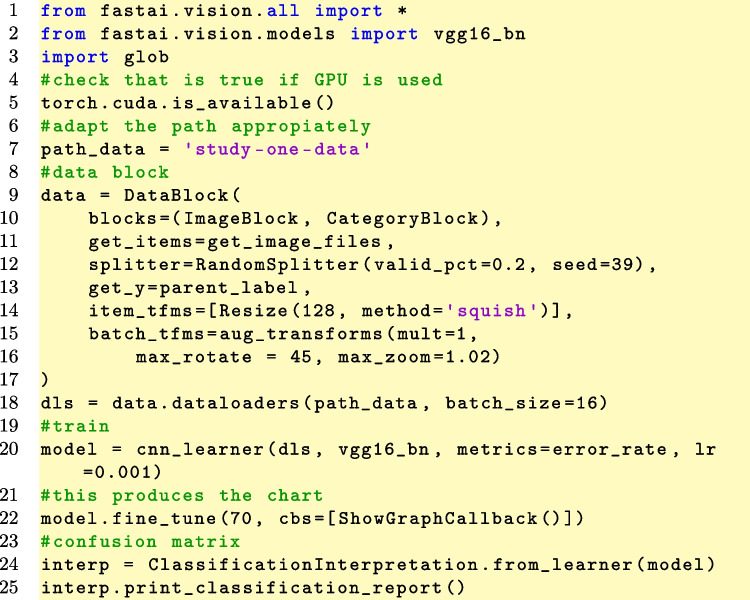
Code employed to replicate study one with the *blinded* version of the dataset. See the Jupyter Notebooks in the following GitHub repository for the output and further details [76]

The life-cycle of an ML model does not finish with the publication or deployment of the solution. It finishes when it is replaced or disposed. Meanwhile, its performance needs to be constantly assessed to detect performance decay (see Fig. 5). The discipline in charge of monitoring ML models was recently named as MLOps [65], and comprises a series of continuous integration practices for each part of the delivered software. MLOps does not just entail software testing (e.g., of routines) and maintenance (e.g., of libraries) but also data operations assessing the quality and state of the data sources and inferences.

Two of the most important events that can damage the system performance include concept drift and data drift. Concept drift events invalidate the relationships between inputs and outputs learnt by the model, i.e., $P(y \vert X)$ changes [66]. For instance, the definition of a class changes over time, requiring re-training to prevent a performance decrease. Conversely, data drift events entail a change in the data distribution of our setting, i.e., a change in *P*(*X*). However, the relationship to the predicted output i.e., $P(y \vert X)$, remains the same. Potential sources of data drift events include the replacement of sensors and data acquisition devices (e.g., machines) as well as external events such as epidemiological or public health changes.

These are just two examples of a wider range of tasks that fall under the umbrella of MLOps. Stirbu et al. provide a detailed approach of continuous design control for industrial medical ML systems [67].

### ML models interpretability

As Goyal et al. note, the decision flows of computer-aided diagnosis solutions often differ from those of clinicians, hampering interpretability and inspection of the results [49] due to the black-box nature of ML models. From a design point of view, dividing the task into several sub-tasks (e.g., (1) detecting pathologies, then (2) diagnosing the disease from the pathologies) can ease both interpretability and maintenance [47].

However, the definition of interpretability in ML varies across authors. For instance, Miller et al define it as the degree to which an observer can understand the cause of a decision [68], while Kim et al. consider a method interpretable “if a user can correctly and efficiently predict the method’s results” [69]. Regardless of the definition nuances, interpretability has recently gained importance as new regulations encode the right to be informed of data subjects, which requires that any information about the processing of personal data is easily accessible and easy to understand, specially regarding automated individual decision-making [70].

In his book “Interpretable Machine Learning”, Chrostoph Molnar provides a detailed guide of the available techniques to assess and improve ML interpretability [71]. They can be separated in *intrinsic* and *post hoc*. The former refers to models whose simple structure allows human interpretation, such as short decision trees or linear regression models. The latter set of methods are conducted after model training. Further, they can be divided in model-specific and model-agnostic methods. Finally, we must differentiate between local (to asess an individual prediction) and global (to audit the entire model behaviour) interpretability. Some global model-agnostic methods include partial dependence (PD) plots and Accumulated Local Effects (ALE) plots which describe how features influence the prediction of a ML model [72]. Nevertheless, for the tasks addressed in this manuscript, we should focus on methods designed for DL due to its success in image classification tasks. For instance, feature attribution methods like SHapley Additive exPlanations (SHAP) plots compute the contribution of each feature to explain a particular prediction [73]. A special case of feature attribution for images is pixel attribution methods, which provide saliency maps indicating the pixels relevant for an image classification instance [74]. Other methods include feature visualisations that help to convey how NNs work [75].

Of course, this paper cannot delve into the previous topics, but we hope the above’s account provides a general overview of the methods relevant to each of the problems addressed in this manuscript.

## Conclusion

To sum up, the general mistake made in the initiator work and other similar works is to believe that naming two folders Chickenpox and Monkeypox and training a classifier with whatever the content is can be enough to build a solution that *actually* distinguishes between Chickenpox and Monkeypox. Unless it is proven otherwise, the more it can said is that the built solution separates the elements from the two folders very well, but we cannot tell whether it is doing so based on the underlying features representative of the disease or not. The presented rebuttal experiment shows that it is not the case.

The second important issue of the subsequent works employing the initiator datasets and other similar datasets is not to review the acquisition process of the datasets employed in their works and ensure that the images were medically relevant for the purpose of building a ML model for computer-aided diagnosis.

The fact that there is no public high-quality Monkeypox dataset does not excuse employing poorly acquired images. A study for proper data acquisition would have to be conducted to acquire and verify Monkeypox, Measles and Chickenpox images in humans. The dataset images lack any coherence or curation. A bad dataset can be worse than no dataset at all. Aforementioned datasets lack medical relevance, which is enormously disappointing given the goals stated in the scientific articles. Simply, because images tagged in stock image repositories are not necessarily medically validated, i.e. they can show pathologies similar to the diseases but without actually corresponding to real cases of such diseases. Moreover, the datasets were not accompanied with metadata or demographics information that could facilitate the evaluation of the models. In this sense, science needs better datasets and incentives for data excellence. After all, building a model to classify aforementioned diseases is not a model’s problem, but rather a data problem [3].

The role of supervisors, reviewers and editors is to ensure the quality of the produced scientific works. In this case, the chain has failed from the very beginning. A dataset claiming to contain images from infected Monkeypox patients calls for institutional review boards (IRB) or ethical committees to assess participants’ consent before publishing or employing such a dataset. A dataset aimed at computer-aided diagnosis requires a clear protocol and medical review of the images to ensure that the quality of the data employed for training Machine Learning models is held up to the highest standards. Finally, researchers employing published datasets must review the acquisition process and ensure the validity of the solutions built with them through diligent external validation [59].

We hope the present paper to draw the attention of researchers and editors for future works. We expect the aforementioned datasets to stop spreading and for new and better datasets to amend the issues of the recent developed solutions. The consequences of poorly trained Machine Learning models in healthcare settings can be disastrous, and we can do better, we must do better.

## Data Availability

Data is available through the referenced papers.

## Appendix: Rebuttal experiment code

Below, we provide a summarised version of the code employed to train the solution for the rebuttal of the results from the original paper. Again, it should be highlighted that the weaknesses of the solution are not the methods in particular, but rather the data that was employed to train the model. The images were manually blinded by adding black rectangles in the parts containing blisters pustules and other rash areas. For further details, the Jupyter Notebooks can be accessed via the following GitHub repository [76].

## Code availability

The code employed for the rebuttal experiment is provided in Appendix in Fig. 6 and it is available in further detail in [76].
